# Feasibility of intercostal high intensity focused ultrasound ablation of clinically relevant volumes under the application of beam shaping

**DOI:** 10.1186/2050-5736-3-S1-O86

**Published:** 2015-06-30

**Authors:** Martijn de Greef, Gerald Schubert, Joost Wijlemans, Julius Koskela, Lambertus W Bartels, Chrit Moonen, Mario Ries

**Affiliations:** 1 University Medical Center Utrecht, Utrecht, Netherlands; 2 Philips Healthcare, Vantaa, Finland

## Background/introduction

Obstruction by the thoracic cage of the high intensity focused ultrasound (HIFU) beam is one of the major challenges in the ablation of abdominal lesions. Several beam shaping methods have been proposed to reduce exposure of the ribs to acoustic energy and to recover focal point intensity. The feasibility of HIFU ablation of a clinically relevant volume applying beam shaping remains however unaddressed. In this study, feasibility was evaluated based on exposure of both the ribs as well as the near-field to acoustic energy. In addition, the volumetric ablation rate was estimated to evaluate whether clinically relevant ablation speeds can be achieved.

## Methods

To establish a safety limit for the exposure of the ribs to acoustic energy, rib heating was studied in an *in vivo* porcine model using MR thermometry (Achieva, Philips Healthcare, Best, the Netherlands). The animals (n = 2) were installed on the Sonalleve V2 HIFU platform (Philips Healthcare, Vantaa, Finland) and sonications (n = 3) were performed for transducer positions where rib obstruction occurred. Ray tracer simulations were used to estimate the corresponding exposure to acoustic energy. Four different sonication geometries were defined (sonication at 30 mm and 50 mm behind rib cage at both 15 mm and 20 mm rib spacing) for which single point sonication and volumetric sonications (circular trajectory with 2mm and 4mm diameter) were simulated. Based on the time required to achieve 65 °C in a defined ROI according to simulation, acoustic energy density at the ribs and the fat – muscle interface was estimated based on ray tracer and ASPW simulations of the acoustic intensity, respectively. Beam shaping was based on rib – ray collision detection and total acoustic power was kept constant. The volumetric ablation rate was estimated based on the ablated volume according to the defined ROI and the inter-sonication waiting time based on a cycle-average energy deposition rate of 100 kJ/h.

## Results and conclusions

Based on MR thermometry, rib temperature increases of 20 – 34 °C were observed at simulated acoustic energy densities of 5.4 – 6.6 J/mm2 (minimum of the 10% surface with the highest exposure). With increasing volume ablated in a single sonication, the near-field energy density at the fat – muscle interface increased with peak levels above 5 J/mm2 in case of sonication with a 4mm diameter circular trajectory (safety limit 2.5 J/mm2). The corresponding rib acoustic energy density in the 1cm2 of rib surface with the highest exposure was < 1 J/mm2 for 30 mm and < 2 J/mm2 for 50 mm sonication depth. For the sonication scenarios for which the near-field energy density was within the specified limits, volumetric ablation rates of ≤ 1 ml/h were estimated. In conclusion, beam shaping by means of the collision detection method is an effective means to protect the ribs against excessive heating. However, by conserving the total acoustic power emitted, this mitigated risk is replaced by the risk of excessive near-field heating. As a consequence, for the particular hardware studied, the volume that can be ablated in a single sonication is required to be limited and this limits the volumetric ablation rate.

